# Pivotal role of myeloid‐derived suppressor cells in infection‐related tumor growth

**DOI:** 10.1002/cam4.6917

**Published:** 2024-03-08

**Authors:** Nozomi Ito, Hironori Tsujimoto, Hiromi Miyazaki, Risa Takahata, Hideki Ueno

**Affiliations:** ^1^ Department of Surgery National Defense Medical College Tokorozawa Japan; ^2^ Division of Biomedical Engineering Research Institute, National Defense Medical College Tokorozawa Japan

**Keywords:** cancer progression, immunosuppression, myeloid‐derived suppressor cells (MDSCs), postoperative infectious complications

## Abstract

**Background:**

In this study, we investigated infection‐related tumor growth, focusing on myeloid‐derived suppressor cells (MDSCs) in clinical and experimental settings.

**Patients and Methods:**

In the clinical study, a total 109 patients who underwent gastrectomy or esophagectomy were included. Blood samples were collected from a preoperative time point through 3 months after surgery, and MDSCs were analyzed using flow cytometry. In animal experiments, peritonitis model mice were created by CLP method. We investigated the number of splenic MDSCs in these mice using flow cytometry. Malignant melanoma cells (B16F10) were inoculated on the back of the mice, and tumor growth was monitored. We compared the level of MDSC infiltration around the tumor and the migration ability between CLP and sham‐operated mice‐derived MDSCs. Finally, we focused on PD‐L1^+^MDSCs to examine the effectiveness of anti‐PD‐L1 antibodies on tumor growth in CLP mice.

**Results:**

In patients with postoperative infectious complication, MDSC number was found to remain elevated 3 months after surgery, when the inflammatory responses were normalized. CLP mice showed increased numbers of MDSCs, and following inoculation with B16F10 cells, this higher number of MDSCs was associated with significant tumor growth. CLP‐mice‐derived MDSCs had higher levels of accumulation around the tumor and had more enhanced migration ability. Finally, CLP mice had increased numbers of PD‐L1^+^MDSCs and showed more effective inhibition of tumor growth by anti‐PD‐L1 antibodies compared to sham‐operated mice.

**Conclusion:**

Long‐lasting enhanced MDSCs associated with infection may contribute to infection‐related tumor progression.

## Graphical abstract

In patients with postoperative infectious complication, MDSCs increased for 3 months post‐surgery when the inflammatory responses were normalized. CLP mice have increased numbers of MDSCs, and tumor inoculation when MDSCs are increased leads to significant tumor growth. Infiltration and migration abilities of CLP mice‐derived MDSCs were higher than those of sham‐operated mice‐derived MDSCs. CLP mice with increased PD‐L1^+^MDSCs showed effective tumor growth inhibition by anti‐PD‐L1 antibody.

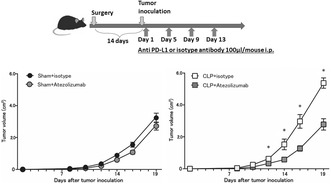

## 1 INTRODUCTION

Although surgical resection of solid tumors is undoubtedly an essential treatment strategy, it often leads to postoperative infectious complications (PICs) and functional disorders.^1^, ^2^, ^3^, ^4^ PIC is known to lead to increases in treatment cost and prolonged hospital stays, and the lack of effective adjuvant therapy is often attributed to PICs.^5^, ^6^ In addition, there is increasing evidence that PIC is associated with unfavorable long‐term outcomes in various malignancies, such as gastric cancer,^7^ colorectal cancer,^8^, ^9^, ^10^ breast cancer,^11^, ^12^ and esophageal cancer.^13^ However, there is no consensus regarding the precise mechanism(s) relating poor long‐term survival to PICs.^14^ We have previously proposed three possible immunological mechanisms involved in PIC‐related tumor progression.^1^ In brief, (i) microbial components may be directly involved in tumor growth, (ii) the mediators released from immunocompetent cells during infection may affect tumor progression, and (iii) suppression of host tumor immunity during infection may result in tumor progression.

It is well known that sepsis or persistent inflammation causes a shift toward an anti‐inflammatory, immunosuppressive state.^15^ Bone et al.^16^ characterized macrophage deactivation, T‐cell anergy, and reduced antigen presentation in this state, which was termed compensatory anti‐inflammatory response syndrome (CARS). Several immunosuppressive properties, such as anti‐inflammatory cytokines, regulatory T cells, and myeloid‐derived suppressor cells (MDSCs), are known to be involved in the development of CARS.^15^, ^17^, ^18^, ^19^, ^20^

Myeloid‐derived suppressor cells were first identified in cancer and have strong immunosuppressive properties,^20^ including the increased expression of programmed cell death protein ligand 1 (PD‐L1) and cytotoxic T‐lymphocyte‐associated protein 4 (CTLA‐4), and actively participate in multiple aspects of tumor progression.^21^, ^22^ Experimental sepsis models revealed a dramatic expansion of MDSCs in the bone marrow, lymph nodes, and spleen.^23^ Interestingly, Delano et al. demonstrated that splenic MDSCs number increased over several months in peritonitis murine models.^24^ In humans, severe sepsis caused an increase in circulating MDSCs that persisted weeks to months after septic insults.^25^ Thus, we hypothesized that long‐lasting enhanced MDSCs caused by PIC may contribute to infection‐related tumor progression. To validate this scenario, we monitored the MDSCs enhanced by infection or PIC in both clinical and experimental settings and investigated the involvement of infection‐related enhanced MDSCs in tumor progression, as well as the efficacy of blocking PD‐1/PD‐L1 pathway in an experimental peritonitis model.

## 2 MATERIALS AND METHODS

### 2.1 Patients

Of the 133 patients who underwent gastrectomy or esophagectomy at the National Defense Medical College hospital between January 2019 and December 2020, 109 patients were included in this study (Tables S1 and S2). Considering the effect of chemotherapy on the dynamics of MDSCs, 24 patients who received adjuvant chemotherapy were excluded. All protocols were approved by the Institutional Review Board of the National Defense Medical College, and written consent was obtained prior to the study. (Permission number: 3002).

### 2.2 Sample collection and definition of PICs

EDTA‐anticoagulated human blood samples were collected preoperatively, at postoperative days (POD) 1 and 7, and 1 and 3 months after surgery. Samples were placed in cold storage, and analysis was performed within 48 h of sample collection. The development of PICs was defined according to the Clavian–Dindo classification, with Grade 2 or higher being classed as PIC^26^ (Table S3).

### 2.3 Flow cytometric analysis of human MDSCs

Human whole blood samples were labeled with fluorescent dye‐conjugated monoclonal antibodies against CD11b‐FITC (clone: ICRF44, Thermo Fisher Scientific, USA), CD33‐PE (clone: D3HL60.251, Beckman Coulter, USA), HLA‐DR‐PC5 (clone: Immu‐357, Beckman Coulter, USA), and CD14‐PC7 (clone: RMO52, Beckman Coulter, USA). Samples were hemolyzed using a lysis buffer and suspended in IsoFlow (Beckman Coulter, USA). Flow cytometric analysis was performed with Cytomics FC500 (Beckman Coulter, USA), and the results were analyzed with Flowjo software (Tree Star, Inc., Ashland, OR). As previously reported, CD11b^+^CD33^+^HLA‐DR^low/‐^CD14^+^ cells were gated and regarded as human MDSCs^25^, ^27^ (Figure S1).

### 2.4 Mice

Male C57BL/6 mice (8–10 weeks old) were purchased from Charles River Laboratories Japan (Kanagawa, Japan) and given food and water ad libitum. All animal procedures were conducted according to protocols approved by the National Defense Medical College Animal Care and Use Committee (Permission number: 17080).

### 2.5 Cell line and culture

B16F10 cells, a murine melanoma cell line, were gifted by Prof. Nariyoshi Shinomiya, National Defense Medical College. Cells were maintained in Roswell Park Memorial Institute (RPMI) 1640 medium containing 10% heat‐inactivated fetal bovine serum (FBS) and antibiotics in an atmosphere of 5% CO_2_ at 37°C.

### 2.6 Animal model

Polymicrobial peritonitis was induced by cecal ligation and puncture as previously described.^28^, ^29^ Briefly, mice were anesthetized with intraperitoneal injection of ketamine (100 mg/kg) and xylazine (10 mg/kg), then the lower quadrants of their abdomen were shaved, and the surgical area was disinfected. A 5 mm midline incision was made. After fascial and peritoneal incision, the cecum was exposed and ligated at two branches from the terminal end of the ileocecal artery (almost 10 mm from the tip of the cecum) with 4–0 silk. Double punctures midway between the ligation site and the tip of the cecum were then performed with a 26‐G needle. The cecum was relocated, and the peritoneum and fascia were closed via a simple running suture with 5–0 monofilament. The skin was also sutured with 4–0 monofilament. One milliliter of saline was administered subcutaneously for fluid resuscitation. Sham‐operated mice underwent the same procedure without cecal ligation and puncture. The survival rate at post‐operation day 14 in this CLP model was 94%.

At 14 or 56 days after surgery, 2 × 10^5^ B16F10 cells/200 μL PBS were subcutaneously inoculated into the back of the mice. Tumor growth was monitored 9, 12, 14, 16, and 19 days after inoculation, and the tumor volume was calculated as 0.5 × length × width × width.

### 2.7 Enzyme‐linked immunosorbent assay

Serum levels of CRP and interleukin (IL)‐6 were measured by an enzyme‐linked immunosorbent assay (ELISA) using the High‐Sensitive C‐Reactive Protein ELISA kit (KAMIYA Biomedical Company, USA) and the BD OptEIA™ ELISA Set (Becton, Dickinson and Company, USA).

### 2.8 Flow cytometric analysis of murine MDSCs

Mice were sacrificed 7, 14, 28, or 56 days after surgery, and the spleens were removed. The collected spleens were hemolyzed to prepare a splenic mononuclear cell suspension (Sp‐MNCs). Sp‐MNCs were labeled with fluorescent dye‐conjugated monoclonal antibodies against CD11b‐FITC (clone: M1/70, Thermo Fisher Scientific, USA), Gr‐1‐PE (clone: RB6‐8C5, Thermo Fisher Scientific), CD274 (B7‐H1)‐PE (clone: MIH5, Thermo Fisher Scientific), Ly‐6C‐APC (clone: HK1.4, Thermo Fisher Scientific), and Ly6G‐APC‐Cyanine7 (clone:1A8, TONBO biosciences, USA). As previously reported, CD11b^+^Gr‐1^+^ cells were gated and regarded as mouse MDSCs.^21^, ^30^, ^31^ CD11b^+^Ly6G^+^Ly6C^low^ cells were defined as granulocytic (G)‐MDSCs and CD11b^+^Ly6G^−^Ly6C^high^ cells were defined as monocytic (M)‐MDSCs, according to the previous report.^19^ Flow cytometric analysis was performed using Cytomics FC500 (Beckman Coulter, USA), and the results were analyzed using Flowjo software (Tree Star, Inc., Ashland, OR).

### 2.9 Isolation and transfer of CLP‐derived MDSCs of mice

Mice were sacrificed 14 days after the CLP operation, and the spleens were removed. The collected spleens were hemolyzed to prepare a Sp‐MNC suspension. Sp‐MNCs were labeled with fluorescent dye‐conjugated monoclonal antibodies against CD11b‐FITC (Thermo Fisher Scientific) and Gr‐1‐PE (Thermo Fisher Scientific). CD11b^+^Gr‐1^+^ cells were isolated with a Cell Sorter SH800 (Sony, Tokyo) for subsequent experiments. Sham‐operated mice underwent transplantation of 8.0 × 10^6^ or 1.0 × 10^6^ CLP mice‐derived MDSCs into the tail vein 14 days after the operation.

### 2.10 Histology and immunostaining

Tumors were harvested and fixed with 4% PFA. Tissues were then embedded in optimal cutting temperature (OCT) compound and frozen in liquid nitrogen. Tissue sections were cut at a thickness of 20 μm. After washing with 1 × PBS, the sections were incubated with 10% goat serum at 4°C overnight, followed by incubation with antibodies against CD11b (clone: ITGAM, Novus Biologicals, USA) at dilution of 1:500 and Ly6G and Ly6C (clone: RB608C5, Abcam, Cambridge, MA, USA) at dilution of 1:500 at 4°C overnight. Subsequently, Alexa Fluor® 488‐conjugated (Abcam) and Alexa Fluor® 594‐conjugated (Molecular Probes, Eugene OR, USA) secondary antibodies were added to slides at room temperature for 1 h. Finally, nuclear staining was performed with 4′,6‐diamidino‐2‐phenylindole (Sigma‐Aldrich, St. Louis, MO, USA). Immunofluorescence images of the stained tissues were taken with a BZ‐X700 microscope (Keyence, Japan). Roundly nucleated CD11b and Ly6G and Ly6C‐positive cells were identified as MDSCs and analyzed with a hybrid cell counting device (BZ‐H4C, Keyence, Japan). At the tumor margin, the number of MDSCs per unit area was measured at 200 times the visual field and at each of 5 locations, and the average value was calculated. Additionally, the tumor area at the maximum cut surface of the tumor was calculated from the hematoxylin‐stained section, and the relationship between the tumor area and the number of MDSCs was examined.

### 2.11 Migration assay

To evaluate the difference in migration ability between CLP and sham‐operated mice‐derived MDSCs, trans‐well assays were performed. B16F10 (2 × 10^4^ cells/well) were seeded in the lower chamber of a 24 trans‐well system (Corning, NY, USA, pore size 8 μm, 24 wells) and cultured for 24 h in RPMI 1640 medium containing 10% heat‐inactivated FBS and antibiotics in an atmosphere of 5% CO_2_ at 37°C. MDSCs were collected from the Sp‐MNCs of CLP and sham‐operated mice 14 days after the operation using a MACS Myeloid‐derived Suppressor Cells Isolation Kit (Miltenyi Biotec, Germany). After the lower chambers of trans‐well system were incubated for 24 h, 1.0 × 10^5^cells of CLP or sham‐operated mice‐derived MDSCs in serum‐free media were added into the upper chamber and cultured for 24 h in an atmosphere of 5% CO_2_ at 37°C. After incubating 24 h, the medium in the lower chamber was removed. 4% PFA was added to the lower chamber to fix and stain the cells with antibodies against Ly6G and Ly6C (clone: RB608C5, Abcam). Finally, nuclear staining was performed with 4′,6‐diamidino‐2‐phenylindole (Sigma‐Aldrich, St. Louis, MO). Immunofluorescence images of the stained tissues were taken with a BZ‐X700 microscope (Keyence, Japan). To collect and count migrating cells, the medium from the lower chamber was stained and analyzed using flow cytometry in the same method as described above.

### 2.12 Administration of PD‐L1 antibody drug to tumor transplanted mice

On day 14 after surgery, 2 × 10^5^ B16F10 cells suspended in 200 μL of PBS were subcutaneously inoculated on the back of the mice. Then, 100 μg/mouse of PD‐L1 antibody (Atezolizumab MPDL3280A, Selleck, USA) or IgG isotype control (Anti‐IgG 609–4103, Rockland, USA) was intraperitoneally administered 4 times every 4 days after tumor inoculation. Tumor growth was monitored 9, 12, 14, 16, and 19 days after inoculation, and the tumor volume was calculated as 0.5 × length × width × width.

### 2.13 Statistical analysis

Statistical analyses were performed using JMP Pro 13.1.0 (SAS Institute Inc., Cary, USA). Results are presented as mean ± standard error of the mean unless stated otherwise. Welch's *t*‐test, Mann–Whitney's *U*‐test, and one‐way analysis of variance (one‐way ANOVA) were performed, and post hoc Tukey procedures were performed where appropriate to correct for multiple comparisons. *p* values of <0.05 were considered statistically significant.

## 3 RESULTS

### 3.1 Inflammatory measures and MDSC number in patients with PIC after gastrointestinal surgeries

The clinical characteristics of patients who underwent gastrectomy or esophagectomy are depicted in Tables S1 and S2, respectively. In patients who underwent gastrectomy, those with PIC had a significantly higher prevalence of diabetes and experienced longer hospital stays than those without PIC (Table S1). In addition, patients with PIC had a larger tumor size, more advanced *T* and *N* factors, a higher rate of laparotomy, and more intraoperative bleeding than those without PIC. In patients who underwent esophagectomy, those with PIC had a significantly lower percentage of previous laparotomy and experienced longer hospital stays than those without PIC. There were no differences in surgical and pathological factors (Table S2).

**FIGURE 1:**
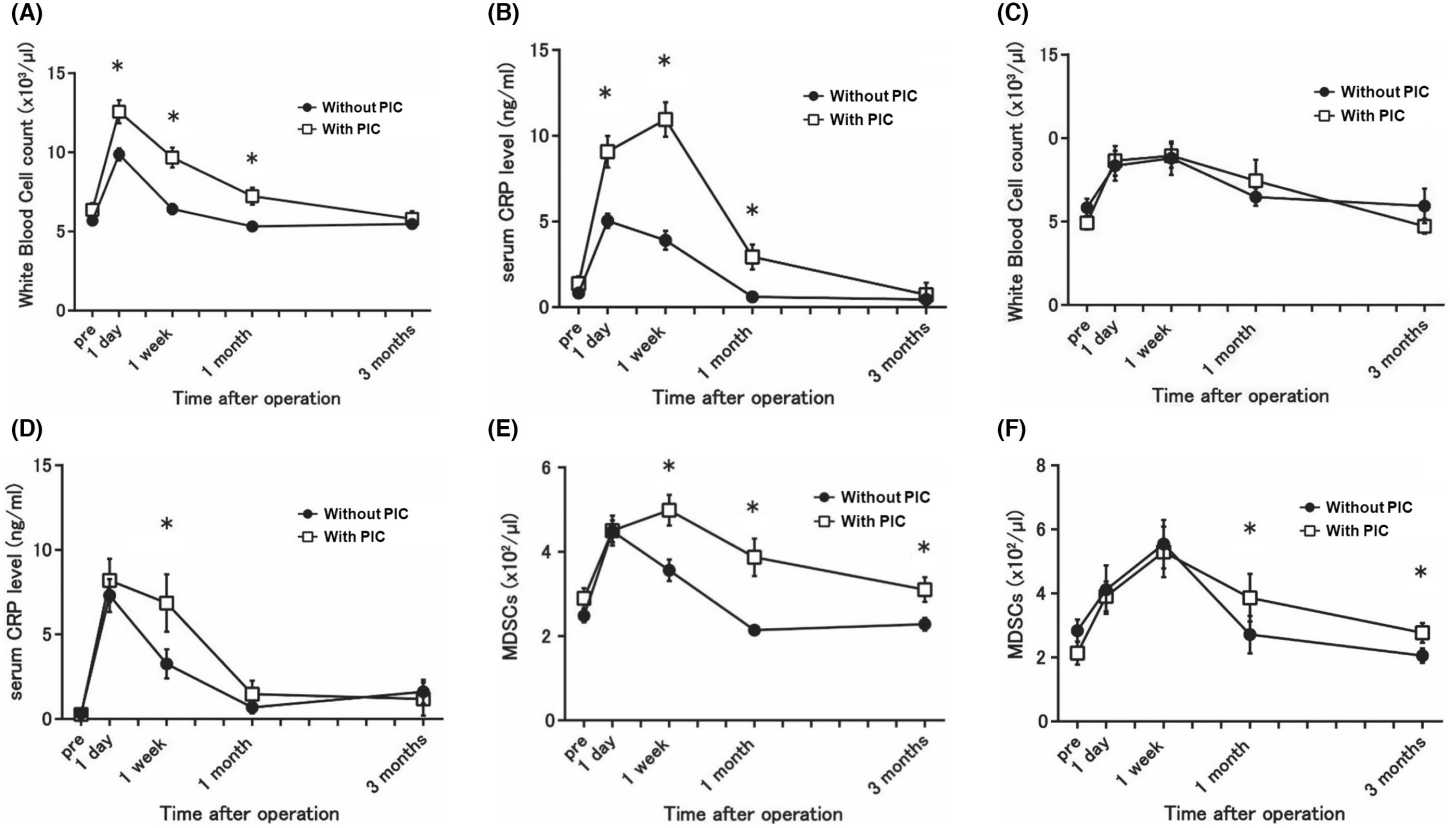
Postoperative changes in inflammatory measures and the number of myeloid‐derived suppressor cells (MDSCs) in patients who underwent gastrectomy or esophagectomy. In patients who underwent gastrectomy, those with PIC had higher WBC counts and higher CRP levels from POD1 to 1 month after gastrectomy. In patients who underwent esophagectomy, there were no differences in postoperative WBC counts or CRP levels, except for CRP levels at 1 week post‐esophagectomy. The number of MDSCs in patients with PIC was significantly higher 1 month after surgery than those without PIC, and MDSC number remained significantly higher for both surgeries even at 3 months post‐surgery when WBC counts and CRP levels were normalized. (A) White blood cell counts after gastrectomy. (B) Serum CRP levels after gastrectomy, (C) White blood cell counts after esophagectomy. (D) Serum CRP levels after esophagectomy. (E) The number of MDSCs after gastrectomy. (F) The number of MDSCs after esophagectomy. *p < 0.05. CRP, C‐reactive protein; MDSCs, myeloid‐derived suppressor cells.

In patients who underwent gastrectomy, those with PIC had higher white blood cells (WBC) counts and higher CRP levels from POD1 to 1 month after gastrectomy (Figure 1A,B). In patients who underwent esophagectomy, there were no differences in postoperative WBC counts or CRP levels, except for CRP levels at 1 week post‐esophagectomy (Figure 1C,D). The number of MDSCs in patients with PIC was significantly higher 1 month after surgery than those without PIC, and MDSC number remained significantly higher for both surgeries even at 3 months post‐surgery when WBC counts and CRP levels were normalized (Figure 1E,F).

### 3.2 Inflammatory responses in CLP mice

**FIGURE 2:**
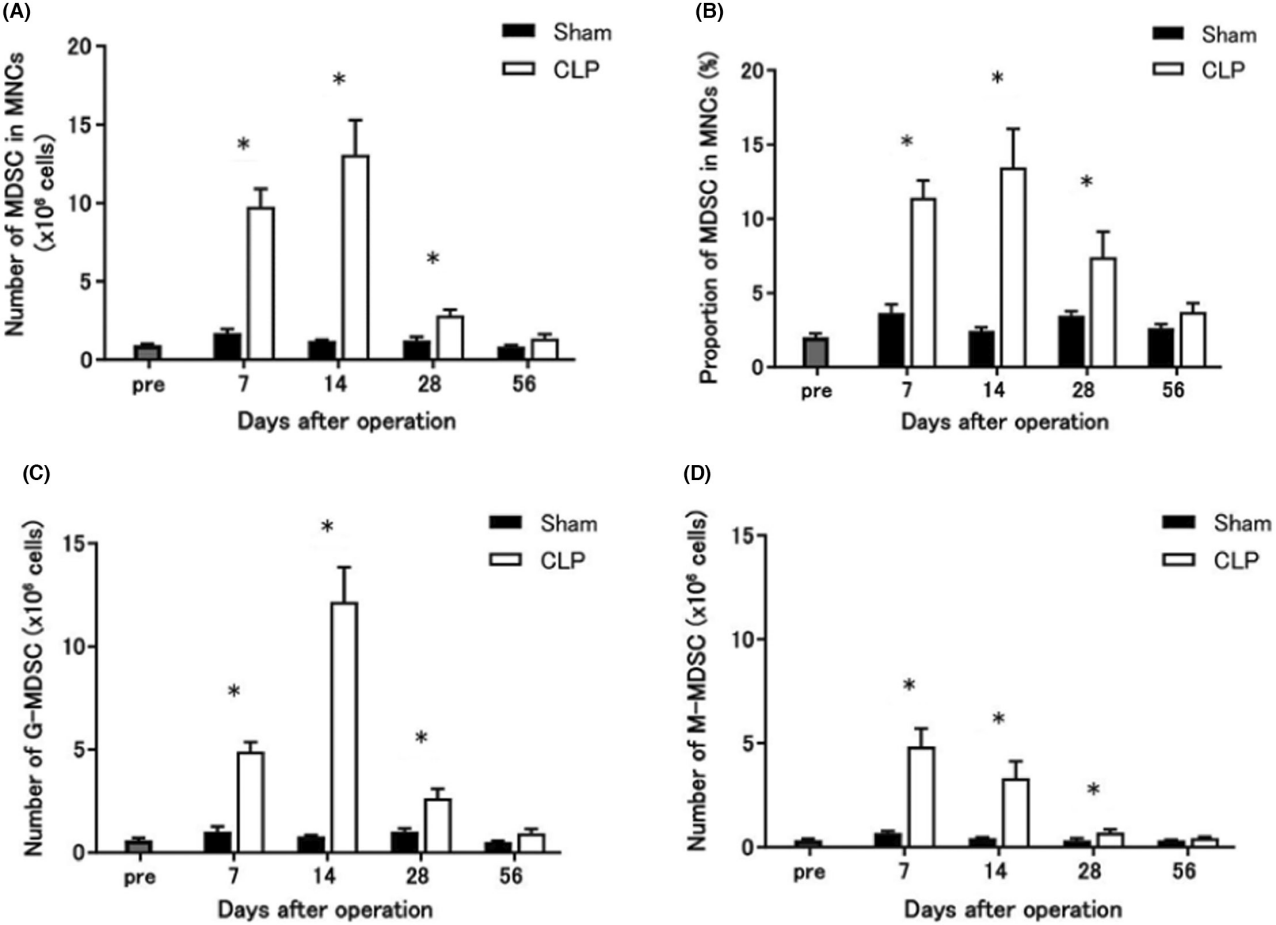
Changes in MDSC numbers after CLP or sham operation. Both the number and proportion of MDSCs in CLP mice were significantly higher than those in sham‐operated mice between 7 and 28 days after surgery. However, by 56 days after surgery, there were no differences in the number and proportion of MDSCs between CLP and sham‐operated mice. The numbers of MDSCs of each subset, G‐MDSCs and M‐MDSCs, were significantly higher in CLP mice than those in sham‐operated mice. The timing of the peak numbers of G‐MDSCs and M‐MDSCs in CLP mice differed between the two subsets. (A) The number of MDSCs after surgery. (B) The proportion of MDSCs in MNCs after surgery. (C) The number of granulocytic MDSCs after surgery. (D) The number of monocytic MDSCs after surgery. All data are represented as mean ± SEM in three independent experiments: *n* = 4 mice per group. *p < 0.05. CLP, cecum ligation and puncture; G‐MDSC, granulocytic myeloid‐derived suppressor cells; MDSCs, myeloid‐derived suppressor cells; M‐MDSC, monocytic myeloid‐derived suppressor cells; MNC, mononuclear cell.

In peritonitis model mice generated by cecal ligation and puncture (CLP), all animals were confirmed to have an intra‐abdominal abscess and splenomegaly at autopsy (Figure S2A). The size of the abscess peaked at 7 days after CLP and then gradually reduced (Figure S2B). The body weight of CLP mice decreased transiently and was significantly lower than that of sham‐operated mice until 28 days after surgery (Figure S2C). The weight of the spleen of CLP mice increased and peaked at 14 days after surgery, and this increase was not observed in sham‐operated mice at either 7 or 14 days after surgery (Figure S2D). Although there were no differences in the WBC counts and CRP levels (Figure S2E,F), the number of splenic mononuclear cells (Sp‐MNCs) in CLP mice was significantly higher than that in sham‐operated mice both 7 and 14 days after surgery (Figure S2G). In addition, the serum interleukin (IL)‐6 level in CLP mice was significantly higher than that in sham‐operated mice both 6 and 24 h after surgery, peaking at 6 h (Figure 2H).

### 3.3 Changes in MDSC number in CLP and sham‐operated mice

Both the number and proportion of MDSCs in CLP mice were significantly higher than those in sham‐operated mice between 7 and 28 days after surgery (Figure 2A,B). However, by 56 days after surgery, there were no differences in the number of splenic mononuclear cells and the number and proportion of MDSCs between CLP and sham‐operated mice. The numbers of MDSCs of each subset, G‐MDSCs and M‐MDSCs, were significantly higher in CLP mice than those in sham‐operated mice. The timing of the peak numbers of G‐MDSCs and M‐MDSCs in CLP mice differed between the two subsets (Figure 2C,D).

### 3.4 Tumor growth in CLP and sham‐operated mice

**FIGURE 3:**
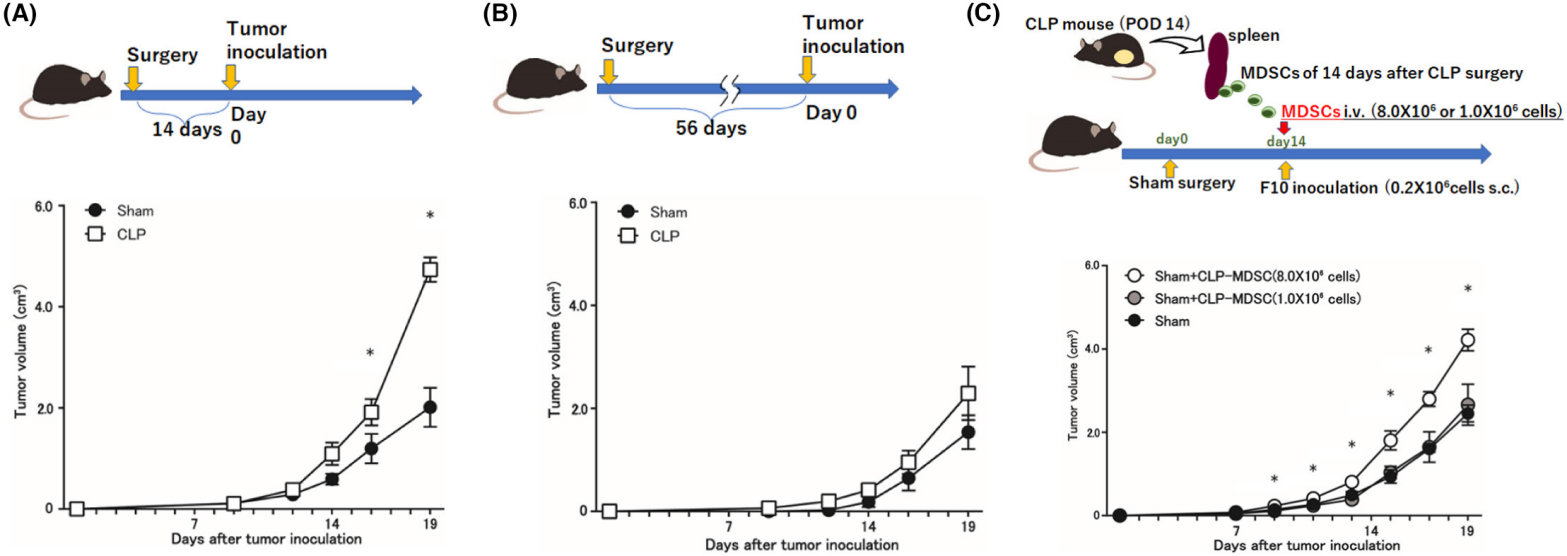
Tumor volume after CLP or sham operation and transplantation of CLP mice‐derived MDSCs following tumor inoculation. (A, B) B16F10 (2.0 × 10^5^ cells/200 μL PBS) were inoculated either 14 days after surgery, when MDSC number had been found to peak, or 56 days after surgery, when the number of MDSCs was no longer different from that in sham‐operated mice. When tumors were inoculated 14 days after surgery (A), tumor growth was significantly enhanced in CLP mice compared to sham‐operated mice. In contrast, when tumors were inoculated 56 days after surgery, there was no statistically significant difference in tumor growth between the two groups (B). (C) High (8.0 × 10^6^ cells/200 μL PBS) or low (1.0 × 10^6^ cells/200 μL PBS) amounts of CLP mice‐derived MDSCs were intravenously injected into sham‐operated mice 14 days after sham operation, and B16F10 cells (2.0 × 10^5^ cells/200 μL PBS) were then subcutaneously inoculated into the back of mice. Injection of high amounts of CLP‐derived MDSCs led to significantly enhanced tumor growth compared to the injection of low amounts of CLP‐derived MDSCs or the degree of tumor growth in mice without MDSC injection. All data are represented as mean ± SEM in three independent experiments: *n* = 4 mice per group. *p < 0.05. CLP, cecum ligation and puncture; MDSCs, myeloid‐derived suppressor cells; POD, post‐operative day.

To investigate the role of enhanced MDSCs in tumor growth in CLP mice, B16F10 (2.0 × 10^5^ cells/200 μL PBS) were inoculated either 14 days after surgery, when MDSC numbers had been found to peak, or 56 days after surgery, when the number of MDSCs was no longer different from that in sham‐operated mice. When tumors were inoculated 14 days after surgery, tumor growth was significantly enhanced in CLP mice compared to sham‐operated mice (Figure 3A). In contrast, when tumors were inoculated 56 days after surgery, there was no statistically significant difference in tumor growth between the two groups (Figure 3B).

### 3.5 Transplantation of CLP mice‐derived MDSCs promoted tumor growth

To investigate the in vivo effect of MDSCs derived from CLP mice on tumor growth, high (8.0 × 10^6^ cells/200 μL PBS) or low (1.0 × 10^6^ cells/200 μL PBS) amounts of CLP mice‐derived MDSCs were intravenously injected into sham‐operated mice 14 days after sham operation, and B16F10 cells (2.0 × 10^5^ cells/200 μL PBS) were then subcutaneously inoculated into the back of mice. Injection of high amounts of CLP‐derived MDSCs led to significantly enhanced tumor growth compared to the injection of low amounts of CLP‐derived MDSCs or the degree of tumor growth in mice without MDSC injection (Figure 3C).

### 3.6 Migration of MDSCs around the tumor

**FIGURE 4:**
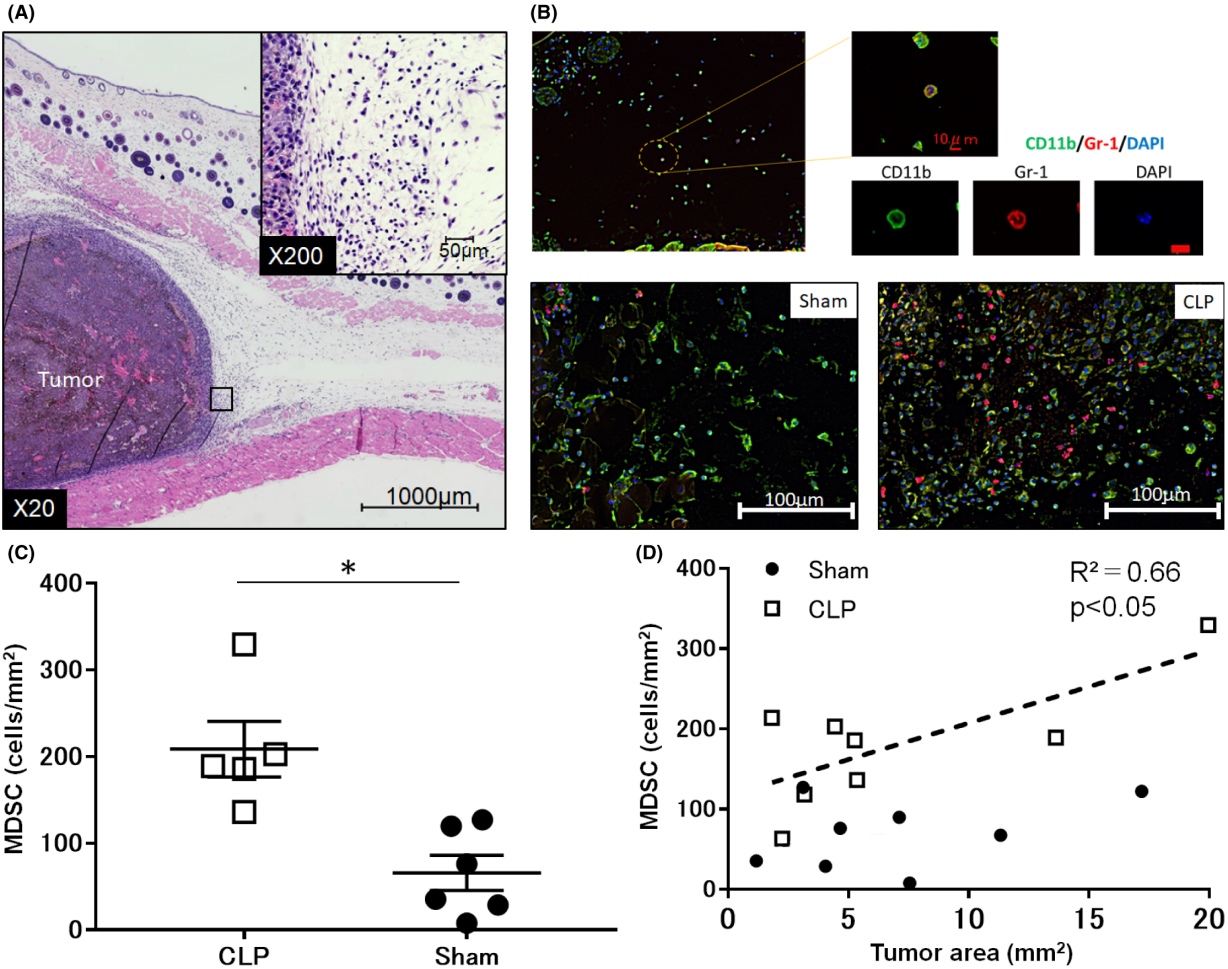
Accumulation of MDSCs around the tumor. Pathological examination showed that various immunocompetent cells infiltrated the tumor 7 days after tumor inoculation. Multiplexed immunofluorescence analysis revealed that CD11b^+^Gr‐1^+^ cells were significantly accumulated around the tumor in CLP mice compared to sham‐operated mice. In addition, there was a positive correlation between the tumor volume and the count of MDSCs around the tumor in CLP mice, while such a correlation was not observed in sham‐operated mice. (A) A representative picture of the area around the tumor 7 days after tumor inoculation (Hematoxylin eosin staining, ×20 and ×200). Scale bars are inserted. (B) A representative picture of the area around the tumor 7 days after tumor inoculation (Multiple fluorescent immunostaining, ×200). Scale bars are inserted. (C) MDSC accumulations around the tumor assessed by multiple fluorescent immunostaining (×200). All data are represented as mean ± SEM in three independent experiments: *n* = 5–6 mice per group. (D) Correlation between tumor volume and the number of accumulated MDSCs. All data are represented as mean ± SEM in three independent experiments: *n* = 5–6 mice per group. *p < 0.05. MDSCs, myeloid‐derived suppressor cells; CLP; cecum ligation and puncture.

To investigate migration of cells around the tumor, pathological examination using Hematoxylin–Eosin (HE) staining and multiplexed immunofluorescence analysis were performed. Pathological examination showed that various immunocompetent cells infiltrated the tumor 7 days after tumor inoculation (Figure 4A). Multiplexed immunofluorescence analysis revealed that CD11b^+^Gr‐1^+^ cells were significantly accumulated around the tumor in CLP mice compared to sham‐operated mice (Figure 4B,C). In addition, there was a positive correlation between the tumor volume and the count of MDSCs around the tumor in CLP mice, while such a correlation was not observed in sham‐operated mice (Figure 4D).

### 3.7 Migration assay on CLP and sham‐operated mice‐derived MDSCs

**FIGURE 5:**
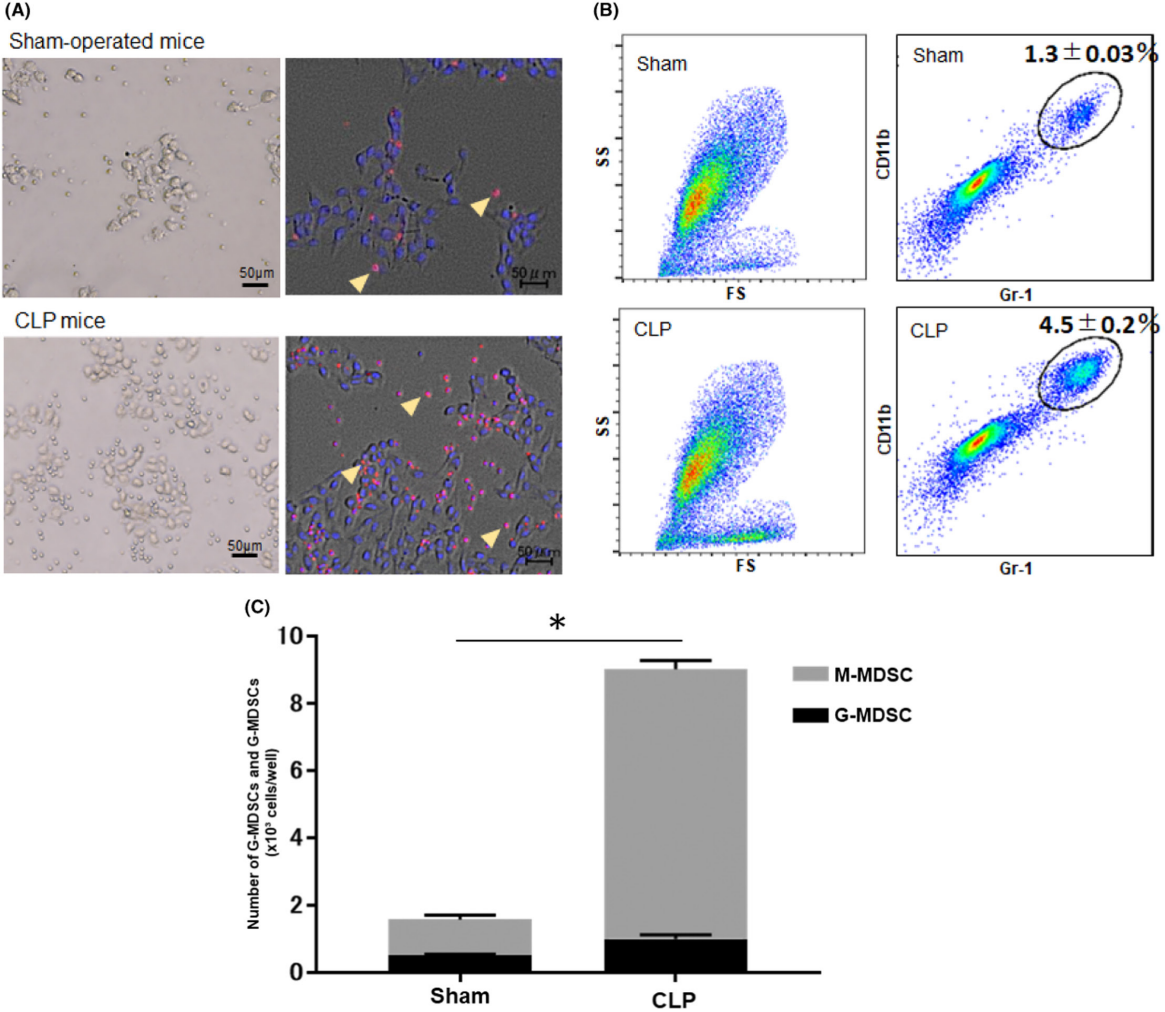
Migration assay of MDSCs to tumor cells. Trans‐well assays were performed to evaluate the difference in migration ability between CLP and sham‐operated mice‐derived MDSCs. Representative pictures of cells at the lower chamber when CLP or sham‐operated mice‐derived. CLP mice‐derived MDSCs were more likely migrate to the lower chamber which was seeded with B16F10 cells than sham‐operated mice‐derived MDSCs. (A) Representative images of the fluorescent immunostaining of the cells taken from the lower chamber after migration assay. Arrow heads indicated Gr‐1 positive cells. Scale bars are inserted. (B) Representative dot plot of flow cytometric analysis. CD11b^+^Gr‐1^+^ cells were regarded as MDSCs. (C) The number of MDSCs migrated to the lower chamber that seeded tumor cells. All data are represented as mean ± SEM in two independent experiments: *n* = 5 mice per group. *p < 0.05. CLP, cecum ligation and puncture; FS, forward scatter; MDSCs, myeloid‐derived suppressor cells; SS, side scatter.

Trans‐well assays were performed to evaluate the difference in migration ability between CLP and sham‐operated mice‐derived MDSCs. Representative pictures of cells at the lower chamber when CLP or sham‐operated mice‐derived MDSCs were added in to the upper chamber are depicted in Figure 5A. CLP mice‐derived MDSCs were more likely migrate to the lower chamber, which was seeded with B16F10 cells, than sham‐operated mice‐derived MDSCs (Figure 5B,C). Among CLP mice‐derived MDSCs, M‐MDSCs had more enhanced migration ability than G‐MDSCs.

### 3.8 PD‐L1 ^+^ MDSCs and the effect of anti‐PD‐L1 antibody on tumor growth

**FIGURE 6:**
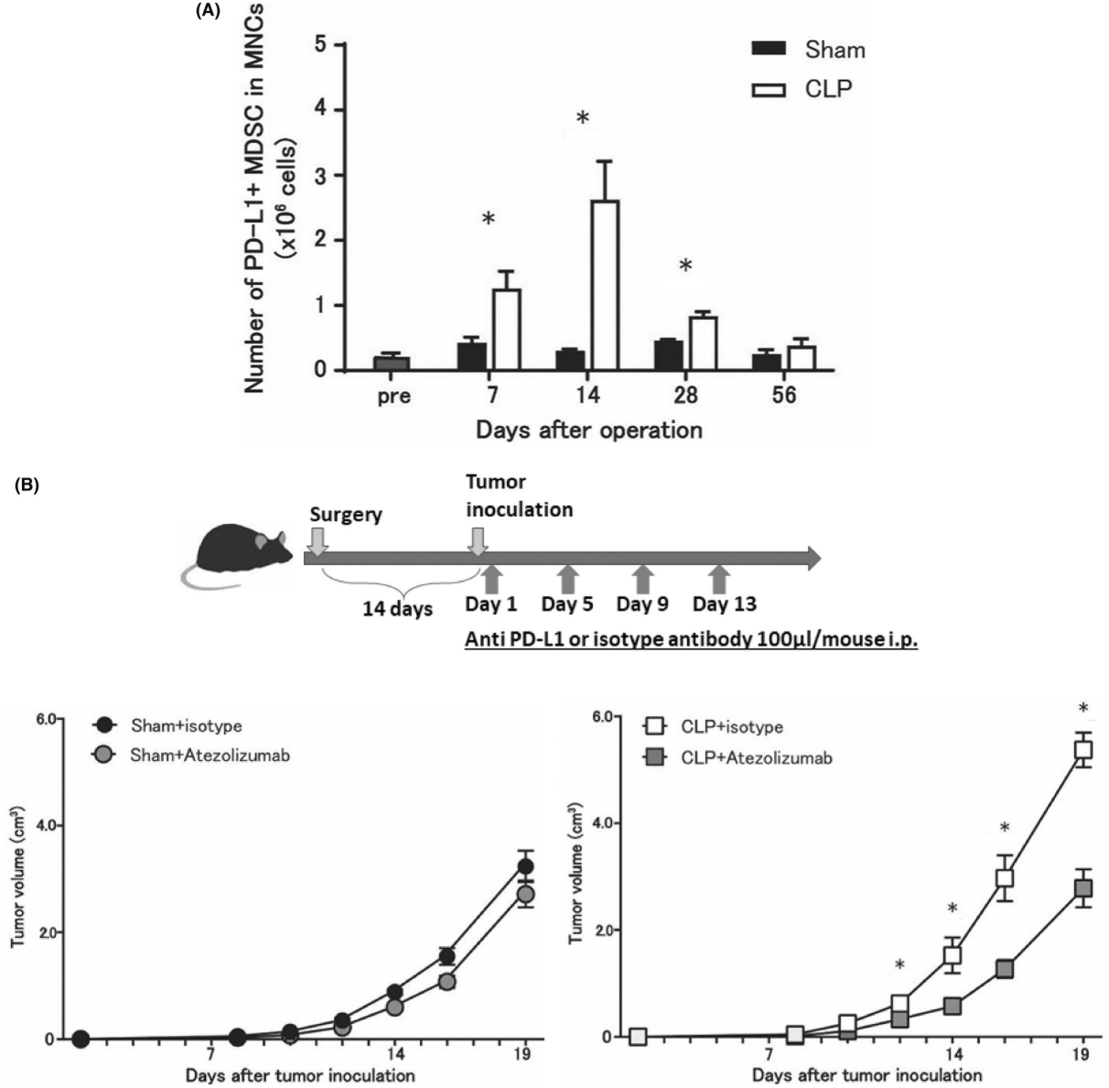
Changes in the number of PD‐L1^+^MDSCs after surgery and the effect of anti‐PD‐L1 antibody on tumor growth. The number of PD‐L1^+^MDSCs in CLP mice was significantly higher than that in sham‐operated mice between 7 and 28 days after surgery. In order to investigate the differences in the effect of blocking PD1/PD‐L1 pathway on tumor growth, sequential intraperitoneal administration of Atezolizumab (anti‐PL‐L1 antibody) or an isotype control was performed. Atezolizumab administration significantly suppressed tumor growth compared to administration of the isotype control in CLP mice; however, such differences were not observed in sham‐operated mice. (A) The number of PD‐L1^+^MDSCs after surgery. All data are represented as mean ± SEM in three independent experiments. *n* = 4 mice per group. (B) The effect of sequential anti‐PD‐L1 antibody administration on tumor growth. All data are represented as mean ± SEM in three independent experiments: *n* = 3–7 mice per group. *p < 0.05. CLP, cecum ligation and puncture; MDSCs, myeloid‐derived suppressor cells; MNCs; mononuclear cells.

The number of PD‐L1^+^MDSCs in CLP mice was significantly higher than that in sham‐operated mice between 7 and 28 days after surgery (Figure 6A). In order to investigate the differences in the effect of blocking PD1/PD‐L1 pathway on tumor growth, sequential intraperitoneal administration of Atezolizumab (anti‐PD‐L1 antibody) or an isotype control was performed (Figure 6B). Atezolizumab administration significantly suppressed tumor growth compared to administration of the isotype control in CLP mice; however, such differences were not observed in sham‐operated mice (Figure 6C).

## 4 DISCUSSION

In this study, we identified a long‐lasting increase in the number of MDSCs in patients with PICs after gastrointestinal surgeries, even after inflammatory measures were normalized in the clinical setting. Furthermore, we showed that CLP‐induced MDSC expansion promoted tumor growth and demonstrated the different effect of anti‐PD‐L1 antibody when administered to an environment with increased numbers of PD‐L1^+^MDSCs, as in CLP mice, compared to its delivery in the absence of infection in the experimental setting.

Myeloid‐derived suppressor cells are immature myeloid cells that show potent immunosuppressive properties and are involved in the formation of immunosuppressive pathologies under inflammation and tumor‐bearing conditions.^22^, ^23^ Accumulating evidence has shown that MDSCs also regulate the immune responses in infections, acute and chronic inflammation, traumatic stress, and sepsis.^32^ Mathias, et al.^25^ reported that enhancements in the number of MDSCs were sustained more than a month in patients with severe sepsis and/or septic shock and that patients with persistently increased percentages of MDSCs had increased nosocomial infections, prolonged ICU stays, and poor functional status at discharge. In this study, we demonstrated that patients with PICs had increased MDSC number for as long as 3 months after gastrectomy or esophagectomy. It is reported that patients with severe sepsis and/or septic shock, even if they survive, have pathophysiologic syndrome of persistent inflammation, immunosuppression, and catabolism, characterized by elevated circulating inflammatory markers, innate immune suppression, lean body mass protein catabolism, and psychological distress for a long time.^33^, ^34^ We previously demonstrated that postoperative pneumonia caused the loss of skeletal muscle volume observed 6 months after surgery and thus the poor prognosis in patients who underwent esophagectomy.^35^ Thus, PICs have more profound long‐term effects on immunological, nutritional, and oncological outcomes than expected.

In this study, significantly enhanced tumor growth was observed in CLP mice when tumors inoculation occurred concurrently with the peak in the number of MDSCs 14 days after CLP; however, such a difference was not seen in 56 days after CLP or sham‐operated mice, in which the number of MDSC decreased. In addition, we demonstrated that transplantation of high amounts of CLP mice‐derived MDSCs enhanced tumor progression compared to transplantation with low amounts of CLP mice‐derived MDSCs or in mice without transplantation. These results suggest that enhancement in the number of MDSCs during infection could, at least in part, be associated with infection‐related tumor growth.

Recent studies have demonstrated that tumor‐associated MDSCs have an important role in tumor progression.^36^, ^37^, ^38^, ^39^ MDSCs are actively recruited to tumor sites, which are regulated by many inflammation‐related cytokines and chemokines produced by tumor cells and other immunocompetent cells.^40^, ^41^ In this study, we demonstrated that MDSCs were significantly accumulated around the tumor in CLP mice compared to sham‐operated mice 7 days after surgery. In addition, CLP mice‐derived MDSCs showed significantly greater ability to migrate to tumor cells compared to sham‐operated mice‐derived MDSCs, and while there was a positive correlation between tumor volume and MDSC accumulation in CLP mice, no correlation was observed in sham‐operated mice. It is reported that circulating MDSCs were found to be significantly increased in cancer patients, and a significant correlation between circulating MDSC and cancer stage has been observed.^42^, ^43^ Wu, et al.^44^ reported that Stage III–IV cervical cancer patients had more tumor‐infiltrating MDSCs than Stage I–II patients. These results suggest that highly activated MDSCs, generated as are result of infection, are more likely to accumulate around the tumor, resulting in enhanced infection‐related local tumor growth.

It is known that MDSCs show a wide range of phenotypes.^22^ In mice, MDSCs can be divided into two subgroups, including G‐MDSC and M‐MDSCs.^30^ Both of these subsets have immunosuppressive functions, but each performs this role through distinct mechanisms.^30^ Briefly, G‐MDSCs inhibit the T cell response primarily through the production of reactive oxygen species (ROS) by antigen‐specific methods,^19^, ^45^ whereas, M‐MDSCs mainly upregulate NO and arginase, produce immunosuppressive cytokines, and inhibit both antigen‐specific and non‐specific T cell responses.^19^, ^30^, ^46^ In this study, there was a difference in the timing of the peak numbers of G‐MDSCs and M‐MDSCs in CLP mice. Additionally, we demonstrated that M‐MDSCs had more enhanced migration ability than G‐MDSCs among CLP mice‐derived MDSCs. Expression of chemokines and chemokine receptors is known to vary by tumor type and subset of MDSCs. Thus, we believe that these differences in migration ability were due to the differential expression of chemokines and/or chemokine receptors among tumor types and subsets of MDSCs.^45^

Immune checkpoint inhibitors targeting the PD‐1/PD‐L1 cascade have been clinically applied to various malignancies.^47^, ^48^, ^49^ However, the PD‐1/PD‐L1 antibody agent alone often has a low response rate of approximately 10%–20%, and there is a lack of effective biomarkers to predict its therapeutic effect.^50^, ^51^ In this study, we demonstrated that PD‐L1^+^MDSCs were significantly increased in CLP mice compared to sham‐operated mice. Furthermore, administration of anti‐PD‐L1 antibody led to a more pronounced inhibitory effect on tumor growth in CLP mice than that in sham‐operated mice. Thus, we believe that targeting PD‐L1^+^MDSCs induced by infection may be an effective treatment strategy in patients with PIC.^21^ For example, patients with PIC may more effectively benefit from adjuvant chemotherapy with anti‐PD‐L1 antibody.^52^

This study has some limitations. In the clinical setting, a relatively small number of patients were included in this study, and the study was also retrospective in nature. It is well known that chemotherapy alters the patient immune status. Among patients who underwent esophagectomy, there was no difference in the transition of MDSC numbers between patients with NAC and without NAC. In patients who underwent gastrectomy, the percentage with pStage II or higher was significantly greater in patients with PIC than in patients without PIC. In Japan, adjuvant chemotherapy is a standard treatment strategy for pStage II/III gastric cancer.^53^ Since chemotherapy is thought to affect the proportion of MDSCs, we excluded patients with adjuvant chemotherapy, which introduced a selection bias.

In conclusion, in patients who developed PIC after gastrectomy or esophagectomy, circulating MDSCs were elevated 3 months after surgery even when other inflammatory measures subsided. The increased number and enhanced migration ability of MDSCs observed in CLP mice appears to promote tumor growth. Thus, long‐lasting enhanced MDSCs associated with infection may contribute to infection‐related tumor progression. In addition, a treatment involving targeting PD‐L1^+^MDSCs may prove to be an effective strategy in patients with PIC.

## AUTHOR CONTRIBUTIONS

**Nozomi Ito:** Conceptualization (lead); data curation (lead); investigation (equal); methodology (equal); supervision (equal); writing – original draft (lead). **Hironori Tsujimoto:** Conceptualization (equal); project administration (equal); supervision (equal); validation (equal); visualization (equal); writing – review and editing (lead). **Hiromi Miyazaki:** Conceptualization (equal); data curation (equal); investigation (equal); methodology (equal); supervision (equal); validation (equal); writing – review and editing (equal). **Risa Takahata:** Conceptualization (equal); funding acquisition (equal); writing – original draft (equal). **Hideki Ueno:** Conceptualization (equal); supervision (equal); writing – review and editing (equal).

## FUNDING INFORMATION

This research did not receive any specific grant from funding agencies in the public, commercial, or not‐for‐profit sectors.

## CONFLICT OF INTEREST STATEMENT

The authors have declared no conflicts of interest.

## ETHICS STATEMENT

For animal experiments, approval was obtained from the National Defense Medical College Animal Care and Use Committee, and for experiments involving humans, approval was obtained from the Institutional Review Board of the National Defense Medical College and written consent was obtained prior to the study.

## CONSENT FOR PUBLICATION

All authors reviewed the manuscript and permitted to submit the manuscript.

## Supporting information


Figure S1.

Figure S2.

Table S1.

Table S2.

Table S3.

## Data Availability

All data are available on the request.
