# Supplementary figures and images for: Pivotal role of myeloid‐derived suppressor cells in infection‐related tumor growth
Source: Cancer Med. 2024 Mar 8;13(4):e6917. doi: 10.1002/cam4.6917 (PMC10923041; doi:10.1002/cam4.6917)

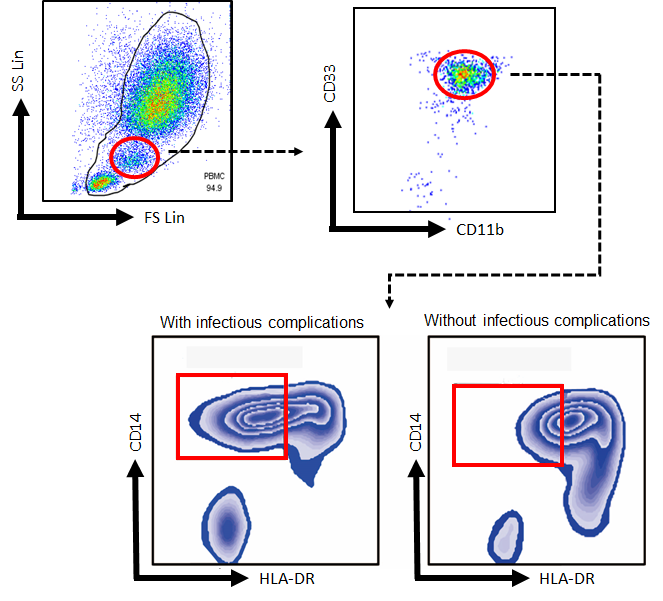

Supplement: Supplementary file 1 — Figure S1. [file CAM4-13-e6917-s004.tif]

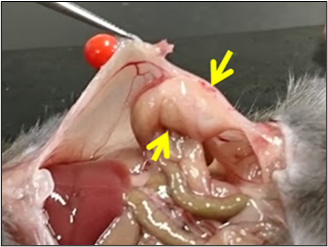

Supplement: Supplementary file 2 — Figure S2. [file CAM4-13-e6917-s001.zip › cam46917-sup-0002-Figure2A.tif]

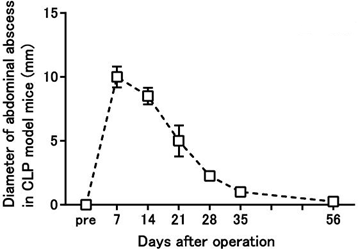

Supplement: Supplementary file 2 — Figure S2. [file CAM4-13-e6917-s001.zip › cam46917-sup-0003-Figure2B.tif]

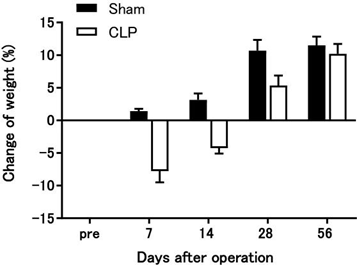

Supplement: Supplementary file 2 — Figure S2. [file CAM4-13-e6917-s001.zip › cam46917-sup-0004-Figure2C.tif]

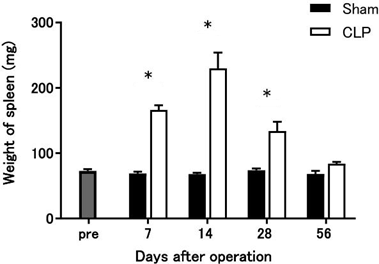

Supplement: Supplementary file 2 — Figure S2. [file CAM4-13-e6917-s001.zip › cam46917-sup-0005-Figure2D.tif]

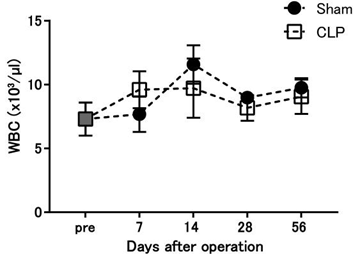

Supplement: Supplementary file 2 — Figure S2. [file CAM4-13-e6917-s001.zip › cam46917-sup-0006-Figure2E.tif]

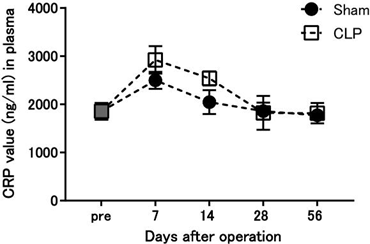

Supplement: Supplementary file 2 — Figure S2. [file CAM4-13-e6917-s001.zip › cam46917-sup-0007-Figure2F.tif]

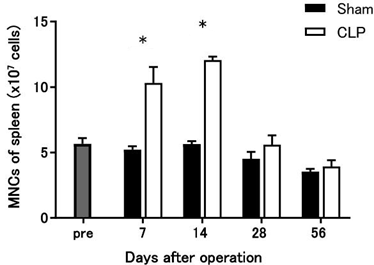

Supplement: Supplementary file 2 — Figure S2. [file CAM4-13-e6917-s001.zip › cam46917-sup-0008-Figure2G.tif]

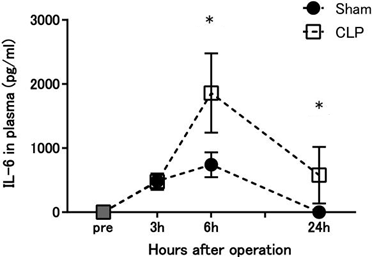

Supplement: Supplementary file 2 — Figure S2. [file CAM4-13-e6917-s001.zip › cam46917-sup-0009-Figure2H.tif]
