# Supplementary material for: Pivotal role of myeloid‐derived suppressor cells in infection‐related tumor growth
Source: Cancer Med. 2024 Mar 8;13(4):e6917. doi: 10.1002/cam4.6917 (PMC10923041; doi:10.1002/cam4.6917)
Supplement: Supplementary file 3 — Table S1. [file CAM4-13-e6917-s005.docx]

sTable 1 Clinicopathological features in patients underwent gastrectomy

| **Factors** | | | **Infectious complications** | | **p-value** |
| --- | --- | --- | --- | --- | --- |
|  |  |  | Yes (N=24) | No (N=57) |  |
| Clinical factors | | |  |  |  |
|  | Age mean (range) | | 76 (53-89) | 72 (46-92) | 0.09 |
|  | Gender | Male | 20 (83%) | 39 (68%) | 0.27 |
|  |  | Female | 4 (17%) | 18 (32%) |  |
|  | Height (cm) mean (range) | | 160.5 (144.6-178.5) | 160.2 (142.2-179.0) | 0.86 |
|  | Weight(kg) mean (range) | | 57.5 (38.0-81.7) | 58.3 (34.7-89.7) | 0.76 |
|  | BMI | | 22.2 (13.7-26.6) | 55.6 (16.0-35.3) | 0.64 |
|  | Co-morbidity |  |  |  |  |
|  | Cardiovascular diseases | Yes | 15 (63%) | 29 (51%) | 0.46 |
|  |  | No | 9 (37%) | 28 (49%) |  |
|  | Diabetes | Yes | 7 (29%) | 5 (9%) | <0.05 |
|  |  | No | 17 (71%) | 52 (91%) |  |
|  | Respiratory diseases | Yes | 4 (17%) | 2 (4%) | 0.06 |
|  |  | No | 20 (83%) | 55 (96%) |  |
|  | Previous laparotomy | Yes | 8 (33%) | 22 (39%) | 0.80 |
|  |  | No | 16 (67%) | 35 (61%) |  |
|  | Hospitalization days median (range) | | 16 (8-225) | 8 (6-44) | <0.05 |
| Surgical factors | | |  |  |  |
|  | Time (min) | | 285 ± 123 | 2641 ± 64 | 0.32 |
|  | Bleeding (g) | | 524 ± 678 | 201 ± 217 | <0.05 |
|  | Surgical approach | Laparotomy | 11 (46%) | 11(19%) | <0.05 |
|  |  | Laparoscopy | 13 (54%) | 46(81%) |  |
|  | Surgical procedure  (Total gastrectomy) | Yes | 9 (38%) | 11(19%) | 0.09 |
|  |  | No | 15 (62%) | 46(81%) |  |
|  | Blood transfusion | Yes | 8 (33%) | 5(9%) | <0.05 |
|  |  | No | 16 (67%) | 52(91%) |  |
| Pathological factors | | | | | |
|  | Localization | U | 7 (29%) | 13 (23%) | 0.59 |
|  |  | M | 8 (33%) | 23 (40%) |  |
|  |  | L | 9 (38%) | 21 (37%) |  |
|  | Macroscopic type | 0 | 6 (25%) | 36 (63%) | <0.05 |
|  |  | 1 | 1 (4％) | 3 (5%) |  |
|  |  | 2 | 7 (29%) | 4 (7%) |  |
|  |  | 3 | 8 (33%) | 10 (18%) |  |
|  |  | 4 | 0 | 1 (2%) |  |
|  |  | 5 | 2 (8%) | 3 (5%) |  |
|  | Diameter (mm) |  | 58 ± 30 | 41 ± 26 | <0.05 |
|  | Pathological type | tub | 12 (50%) | 27 (47%) | 0.16 |
|  |  | por | 11 (46%) | 19 (33%) |  |
|  |  | sig | 0 | 5 (9%) |  |
|  |  | other | 1 (4％) | 6 (11%) |  |
|  | Tumor depth | T1 | 5 (21%) | 34 (60%) | <0.05 |
|  |  | T2 | 5 (21%) | 11 (19%) |  |
|  |  | T3 | 7 (29%) | 10 (18%) |  |
|  |  | T4 | 7 (29%) | 2 (3%) |  |
|  | Nodal metastasis | N0 | 10 (42%) | 41 (72%) | <0.05 |
|  |  | N1 | 4 (17%) | 12 (21%) |  |
|  |  | N2 | 6 (25%) | 3 (5%) |  |
|  |  | N3 | 4 (17%) | 1 (2%) |  |
|  | Pathological stage | Stage I | 8 (33%) | 40 (70%) | <0.05 |
|  |  | Stage II | 5 (21%) | 13 (23%) |  |
|  |  | Stage III | 11 (46%) | 4 (7%) |  |

U; upper third of the stomach, M; middle part of the stomach, L; lower third of the stomach, tub; tubular adenocarcinoma, por; poorly adenocarcinoma, sig; signet-ring cell carcinoma
