# Supplementary material for: Pivotal role of myeloid‐derived suppressor cells in infection‐related tumor growth
Source: Cancer Med. 2024 Mar 8;13(4):e6917. doi: 10.1002/cam4.6917 (PMC10923041; doi:10.1002/cam4.6917)
Supplement: Supplementary file 4 — Table S2. [file CAM4-13-e6917-s002.docx]

sTable 2 Clinicopathological features in patients underwent esophagectomy

| **Factors** | | | **Infectious complications** | | **p-value** |
| --- | --- | --- | --- | --- | --- |
|  |  |  | Yes (N=14) | No (N=14) |  |
| Clinical factors | | |  |  |  |
|  | Age mean (range) | | 70 (48-80) | 74 (65-82) | 0.11 |
|  | Gender | Male | 12 (86%) | 10(71%) | 0.64 |
|  |  | Female | 2 (14%) | 4 (29%) |  |
|  | Height (cm) | | 163 (145-178) | 160 (145-178) | 0.24 |
|  | Weight (kg) | | 59 (33-82) | 54 (37-69) | 0.16 |
|  | BMI | | 21.9 (15.6-25.8) | 21.1 (14.2-25.9) | 0.24 |
|  | Co-morbidity |  |  |  |  |
|  | Cardiovascular diseases | Yes | 7 (50 %) | 8 (57%) | 0.70 |
|  |  | No | 7 (50 %) | 6 (43%) |  |
|  | Diabetes | Yes | 0 | 3(21%) | 0.07 |
|  |  | No | 14 (100%) | 11 (79%) |  |
|  | Respiratory diseases | Yes | 3 (21%) | 1 (7%) | 0.28 |
|  |  | No | 11 (79%) | 13 (93%) |  |
|  | Previous laparotomy | Yes | 1 (7%) | 6 (43%) | <0.05 |
|  |  | No | 13 (93%) | 8 (57%) |  |
|  | Neoadjuvant chemotherapy | No | 4 (29%) | 4 (29%) | >0.99 |
|  |  | Yes | 10 (71%) | 10 (71%) |  |
|  | Hospitalization days median (range) | | 26 (11-59) | 16 (9-26) | <0.05 |
| Surgical factors | | |  |  |  |
|  | Time (min) mean (range) | | 461 ± 24 | 439 ± 24 | 0.25 |
|  | Bleeding (g) mean (range) | | 182 ± 52 | 169 ± 52 | 0.42 |
|  | Thoracic surgery | Thoracoscopy | 14 (100%) | 14 (100%) |  |
|  |  | Thoracotomy | 0 | 0 |  |
|  | Blood transfusion | Yes | 0 | 1 (7%) | 0.30 |
|  |  | No | 14 (100%) | 13 (93%) |  |
| Pathological factors | | | | | |
|  | Localization | Ut | 1 (7%) | 0 | 0.25 |
|  |  | Mt | 7 (50%) | 10 (71%) |  |
|  |  | Lt | 6 (43%) | 4 (29%) |  |
|  | Macroscopic type | 0 | 5 (36％) | 3 (21%) | 0.72 |
|  |  | 1 | 0 | 0 |  |
|  |  | 2 | 2 (14%) | 3 (21%) |  |
|  |  | 3 | 3 (21%) | 5 (36％) |  |
|  |  | 5 | 4 (29%) | 3 (21%) |  |
|  | Diameter (mm) |  | 32 ± 19 | 45 ± 15 | 0.51 |
|  | Tumor depth | T0 | 1 (7%) | 1 (7%) | 0.68 |
|  |  | T1 | 6 (43%) | 3 (21%) |  |
|  |  | T2 | 1 (7%) | 1 (7%) |  |
|  |  | T3 | 6 (43%) | 8 (57%) |  |
|  |  | T4 | 0 | 1 (7%) |  |
|  | Nodal metastasis | N0 | 5 (36％) | 6 (43%) | 0.08 |
|  |  | N1 | 5 (36％) | 0 |  |
|  |  | N2 | 4 (29%) | 5 (36％) |  |
|  |  | N3 | 0 | 2 (14%) |  |
|  |  | N4 | 0 | 1 (7%) |  |
|  | Pathological stage | Stage 0 | 2 (14%) | 1 (7%) | 0.78 |
|  |  | Stage I | 2 (14%) | 2 (14%) |  |
|  |  | Stage II | 5 (36％) | 4 (29%) |  |
|  |  | Stage III | 5 (36％) | 6 (43%) |  |
|  |  | Stage IV | 0 | 1 (7%) |  |

Ut; upper thoracic, Mt; middle thoracic, Lt; lower thoracic
