# Supplementary material for: Pivotal role of myeloid‐derived suppressor cells in infection‐related tumor growth
Source: Cancer Med. 2024 Mar 8;13(4):e6917. doi: 10.1002/cam4.6917 (PMC10923041; doi:10.1002/cam4.6917)
Supplement: Supplementary file 5 — Table S3. [file CAM4-13-e6917-s003.docx]

sTable 3 Incidence of postoperative infectious complications

| Postoperative infectious complications | Number of cases (incidence) | | | | |
| --- | --- | --- | --- | --- | --- |
|  | Esophagectomy N＝14 | |  | Gastrectomy N=28 | |
| Anastomotic leakage | 7 | 25.0% |  | 5 | 6.1% |
| Pneumonia | 7 | 25.0% |  | 2 | 2.4% |
| Pyothorax | 2 | 7.1% |  | 1 | 1.2% |
| Pancreatic fistula | 1 | 3.5% |  | 8 | 9.8% |
| Catheter-related infection | 1 | 3.5% |  | 1 | 1.2% |
| Cholecystitis/cholangitis |  |  |  | 3 | 3.7% |
| Sepsis |  |  |  | 3 | 3.7% |
| Intraperitoneal abscess |  |  |  | 5 | 6.1% |
| Intestinal ischemia/necrosis |  |  |  | 2 | 2.4% |
